# Phase 1 study to evaluate the effects of rifampin on pharmacokinetics of pevonedistat, a NEDD8-activating enzyme inhibitor in patients with advanced solid tumors

**DOI:** 10.1007/s10637-022-01286-8

**Published:** 2022-08-06

**Authors:** Xiaofei Zhou, Ulka Vaishampayan, Devalingam Mahalingam, R. Donald Harvey, Ki Young Chung, Farhad Sedarati, Cassie Dong, Douglas V. Faller, Karthik Venkatakrishnan, Neeraj Gupta

**Affiliations:** 1 Takeda Development Center Americas, Inc. (TDCA), Lexington, MA USA; 2 Karmanos Cancer Institute, Detroit, MI USA; 3 University of Michigan, Ann Arbor, MI USA; 4 Northwestern Medical Group, Chicago, IL USA; 5 Emory University, Atlanta, GA USA; 6 Prisma Health Cancer Institute/ITOR, Greenville, SC USA; 7 EMD Serono Research & Development Institute, Inc., MB Billerica, USA

**Keywords:** Pevonedistat, Rifampin, Advanced malignancies, Pharmacokinetics [4 to 6 max]

## Abstract

**Summary:**

Pevonedistat (TAK-924/MLN4924) is an investigational small molecule inhibitor of the NEDD8-activating enzyme that has demonstrated clinical activity across solid tumors and hematological malignancies. Here we report the results of a phase 1 study evaluating the effect of rifampin, a strong CYP3A inducer, on the pharmacokinetics (PK) of pevonedistat in patients with advanced solid tumors (NCT03486314). Patients received a single 50 mg/m^2^ pevonedistat dose via a 1-h infusion on Days 1 (in the absence of rifampin) and 10 (in the presence of rifampin), and daily oral dosing of rifampin 600 mg on Days 3–11. Twenty patients were enrolled and were evaluable for PK and safety. Following a single dose of pevonedistat at 50 mg/m^2^, the mean terminal half-life of pevonedistat was 5.7 and 7.4 h in the presence and in the absence of rifampin, respectively. The geometric mean AUC_0–inf_ of pevonedistat in the presence of rifampin was 79% of that without rifampin (90% CI: 69.2%–90.2%). The geometric mean C_max_ of pevonedistat in the presence of rifampin was similar to that in the absence of rifampin (96.2%; 90% CI: 79.2%–117%). Coadministration of pevonedistat with rifampin, a strong metabolic enzyme inducer, did not result in clinically meaningful decreases in systemic exposures of pevonedistat. The study results support the recommendation that no pevonedistat dose adjustment is needed for patients receiving concomitant CYP3A inducers.

**ClinicalTrials.gov identifier:**

NCT03486314.

**Supplementary Information:**

The online version contains supplementary material available at 10.1007/s10637-022-01286-8.

## Introduction

Pevonedistat (TAK-924/MLN4924) is an investigational small-molecule inhibitor of the neural precursor cell-expressed, developmentally down-regulated 8 (NEDD8)-activating enzyme (NAE) [1, 2]. NAE conjugates NEDD8 to cullin-RING ligases (CRLs), which control ubiquitination and proteasomal degradation of substrates involved in cell cycle progression, DNA replication, oxidative response and response to hypoxia (HIF1a) [3, 4]. The conjugation of NEDD8 to CRLs – neddylation – activates CRLs to ubiquitinate and degrade their substrates [5]. Literature suggests that neddylation has a pivotal role in regulating immune cell function and tumor angiogenesis in the tumor microenvironment [6]. Pevonedistat forms a covalent adduct with NEDD8 that binds to NAE, preventing neddylation, which subsequently leads to apoptotic cell death [7].

Pevonedistat has demonstrated antitumor activities across a variety of tumor cell lines including solid tumors (colon, lung) and hematological malignancies (myeloma, lymphoma) [7–9]. The activity of pevonedistat as monotherapy or in combination with standard-of-care agents has been evaluated in multiple tumor types, including advanced solid tumors, melanoma, acute myeloid leukemia, myelodysplastic syndromes, multiple myeloma, and lymphoma [2, 10–13]. Clinical studies in patients with solid tumors or hematological malignancies evaluated a range of pevonedistat doses and regimens [10–13]. The maximum tolerated dose (MTD) of pevonedistat was determined to be 25 mg/m^2^ in combination with docetaxel, 20 mg/m^2^ in combination with carboplatin plus paclitaxel, and 20 mg/m^2^ in combination with azacitidine. The recommended dose of pevonedistat for clinical investigation as a single agent was 50 mg/m^2^ administered on Days 1, 3 and 5 in 21-day cycles.

In a clinical mass balance study, pevonedistat was shown to undergo extensive biotransformation following intravenous administration [14], with preferential distribution in whole blood with a whole-blood-to-plasma ratio of 40. The presence of circulating metabolites, the minor contribution of renal clearance to pevonedistat disposition, and the predominantly urinary and fecal elimination of metabolites suggested that hepatic metabolism plays a major role in the overall clearance of pevonedistat. In a population pharmacokinetics (PK) analysis using data from 335 patients across 6 clinical studies, pevonedistat PKs were linear over the dose range of 25–278 mg/m^2^ [15]. Age, sex, tumor type, mild or moderate renal impairment (creatinine clearance ≥ 30 mL/min) had no impact on pevonedistat PK. Body surface area (BSA) was identified as a clinically important source of pevonedistat PK variability, supporting the use of BSA-based dosing.

CYP3A was indicated to play a major role in pevonedistat elimination pathways based on in vitro metabolism (Takeda data on file) and the clinical mass balance study [14]. However, co-administration with the strong CYP3A inhibitor itraconazole did not result in clinically meaningful increase in pevonedistat systemic exposure [16]. While strong CYP3A inhibition does not alter pevonedistat PK, the possibility of clinically meaningful reduction in pevonedistat exposure could not be ruled out. There are examples where a relatively minor effect of CYP3A inhibitors may still be consistent with a meaningful reduction in substrate exposure upon strong induction [17, 18]. Here we present results from a study investigating the effects of rifampin, a strong metabolic inducer of pregnane X receptor (PXR)-inducible drug-metabolizing enzymes (e.g., CYP3A) on pevonedistat PK. Since pevonedistat is a cytotoxic agent and cannot be administered to healthy subjects, this study was conducted in patients with advanced solid tumors. Considering concomitant medications are administered to patients with advanced cancer, this study was designed to inform the risk assessment for potential drug-drug interactions with concomitant use of strong CYP3A inducers during the clinical development of pevonedistat.

## Methods

### Study design

This was an open-label, multi-center study in adult patients with advanced solid tumors (NCT03486314). Eligible patients received a single 50 mg/m^2^ pevonedistat dose via a 1-h infusion on Days 1 and 10, and daily oral dosing of rifampin 600 mg Days 3–11. Serial blood samples for the measurement of pevonedistat plasma concentrations were collected over 48 h following pevonedistat dosing. Pevonedistat PK parameters and ratios of geometric means for maximal plasma concentration (C_max_) and area under the plasma concentration versus time curve from time 0 to infinity (AUC_0–inf_) were calculated in the presence of rifampin (Day 10) and in reference to the absence of rifampin (Day 1), with associated 90% confidence intervals (CI) estimated using analysis of variance.

### Study objectives

The primary objective was to assess the effect of multiple-dose administration of rifampin on the single-dose PK of pevonedistat in adult patients with advanced solid tumors. The secondary objectives were to further characterize pevonedistat PK, safety and tolerability following a single dose at 50 mg/m^2^ in the absence or presence of rifampin following a single intravenous dose at 50 mg/m^2^ in the absence or presence of rifampin.

### Patients

Patients aged ≥ 18 years were required to have histologically or cytologically confirmed metastatic or locally advanced and incurable solid tumors that had progressed despite prior standard therapy or for which conventional therapy was not considered effective. Patients had an Eastern Cooperative Oncology Group (ECOG) performance status of 0 or 1 and expected survival of at least 3 months. Key exclusion criteria included receiving CYP3A inducers within 2 weeks before the first dose of study drug, treatment with any systemic antineoplastic therapy or any investigational products within 21 days before the first dose of study treatment, or antibiotic therapy, radiotherapy, or major surgery within 14 days. Full eligibility criteria are included in the Supplementary Methods.

### Assessments

Serial blood samples for measuring pevonedistat concentrations were collected at intervals from 0 (predose) to 48 h after the single doses of pevonedistat on Day 1 and Day 10. Plasma samples were analyzed for pevonedistat concentrations using a previously reported validated liquid chromatography/mass spectrometry (LC–MS/MS) assay [12].

Safety was assessed by incidence and severity of treatment-emergent adverse events (TEAEs), vital signs, physical examinations, electrocardiograms, and clinical laboratory tests, and toxicities were graded according to the National Cancer Institute Common Terminology Criteria for Adverse Events (NCI CTCAE), version 4.03.

### Pharmacokinetic analysis

Pevonedistat PK parameters were calculated by noncompartmental analysis of the pevonedistat plasma concentration–time data using Phoenix WinNonlin version 8.1 (Certara, Princeton, NJ). The following single-dose PK parameters were derived: C_max_, AUC_0-inf_, AUC from time 0 to the last quantifiable concentration (AUC_0–last_), terminal disposition phase half-life (t_1/2_), clearance (CL), and volume of distribution at steady state (V_ss_).

### Statistical analysis

Pevonedistat PK was assessed in all patients who received the protocol-specified pevonedistat and rifampin doses in Part A, and had sufficient concentration–time data to permit reliable estimation of PK parameters (PK-evaluable population). PK data were summarized using descriptive statistics. For assessment of the effect of rifampin on pevonedistat PK, the ratios of geometric mean C_max_, AUC_last_ and AUC_inf_ of pevonedistat in the presence of rifampin on Day 10 versus in the absence of rifampin on Day 1 and associated 90% CI were calculated based on analysis of variance. Estimates for each PK parameter were obtained using a mixed effects model of log(PK parameter) with fixed terms for the rifampin effect and random terms for patient. Safety was assessed in all patients who received at least one dose of pevonedistat, and TEAEs were coded according to the Medical Dictionary for Regulatory Activities (MedDRA), version 20.0.

## Results

### Patients

A total of 20 patients were enrolled in the study. The median age was 63 years, 50% were male, 75% were white, and the mean weight was 87 kg (Table 1). All patients received at least one dose of pevonedistat and completed protocol-specified dosing and PK sampling requirements, thus were included in the safety and PK-evaluable populations.

**Table 1:** Demographics and baseline disease characteristics (safety population)

	Patients (*N* = 20)
Age, years	
Mean (SD) Median (range)	64.9 (8.7) 63.0 (53–80)
Gender, *n* (%)	
Male Female	10 (50) 10 (50)
Race, *n* (%)	
White Others	15 (75) 5 (25)
Weight, kg^a^	
Mean (SD) Median (range)	87.1 (22.7) 83.5 (51.3–132.9)
BSA, m^2a^	
Mean (SD) Median (range)	2.03 (0.307) 2.01 (1.5–2.6)
Disease type, *n* (%)	
Bile duct cancer	1 (5.0)
Breast cancer – Her2/Neu positive and ER or PR positive (female)	1 (5.0)
Breast cancer – triple negative (female)	1 (5.0)
Breast cancer – Her2/Neu negative (female)	1 (5.0)
GE junction	1 (5.0)
Gastric cancer – Her2-Neu negative	1 (5.0)
Gastroesophageal	1 (5.0)
Intraocular melanoma	1 (5.0)
NSCLC – squamous cell carcinoma	1 (5.0)
Pancreatic cancer (adenocarcinoma)	3 (15.0)
Prostate cancer	2 (10.0)
Retroperitoneal leiomyosarcoma	1 (5.0)
Small cell urothelial carcinoma of the renal pelvis	1 (5.0)
Squamous cell anal cancer	1 (5.0)
Squamous neck cancer with occult primary – metastatic (head and neck cancer)	1 (5.0)
Uterine cancer – endometrial	2 (10.0)
Disease stage, *n* (%)	
IV	20 (100)
Time since initial diagnosis (months)	
* n*	20
Mean (SD)	42.95 (39.297)
Median	25.50
Minimum, maximum	9.0, 141.0
ECOG status at screening (*n* [%])	
0	6 (30.0)
1	14 (70.0)

*BSA* body surface area, *ECOG* Eastern Cooperative Oncology Group, *ER* estrogen receptor, *GE* gastroesophageal, *NSCLC* non-small cell lung cancer, *PR* progesterone receptor, *SD* standard deviation

^a^Baseline measurements for weight and BSA were taken at Day –1

### Pharmacokinetics

**Fig. 1:**
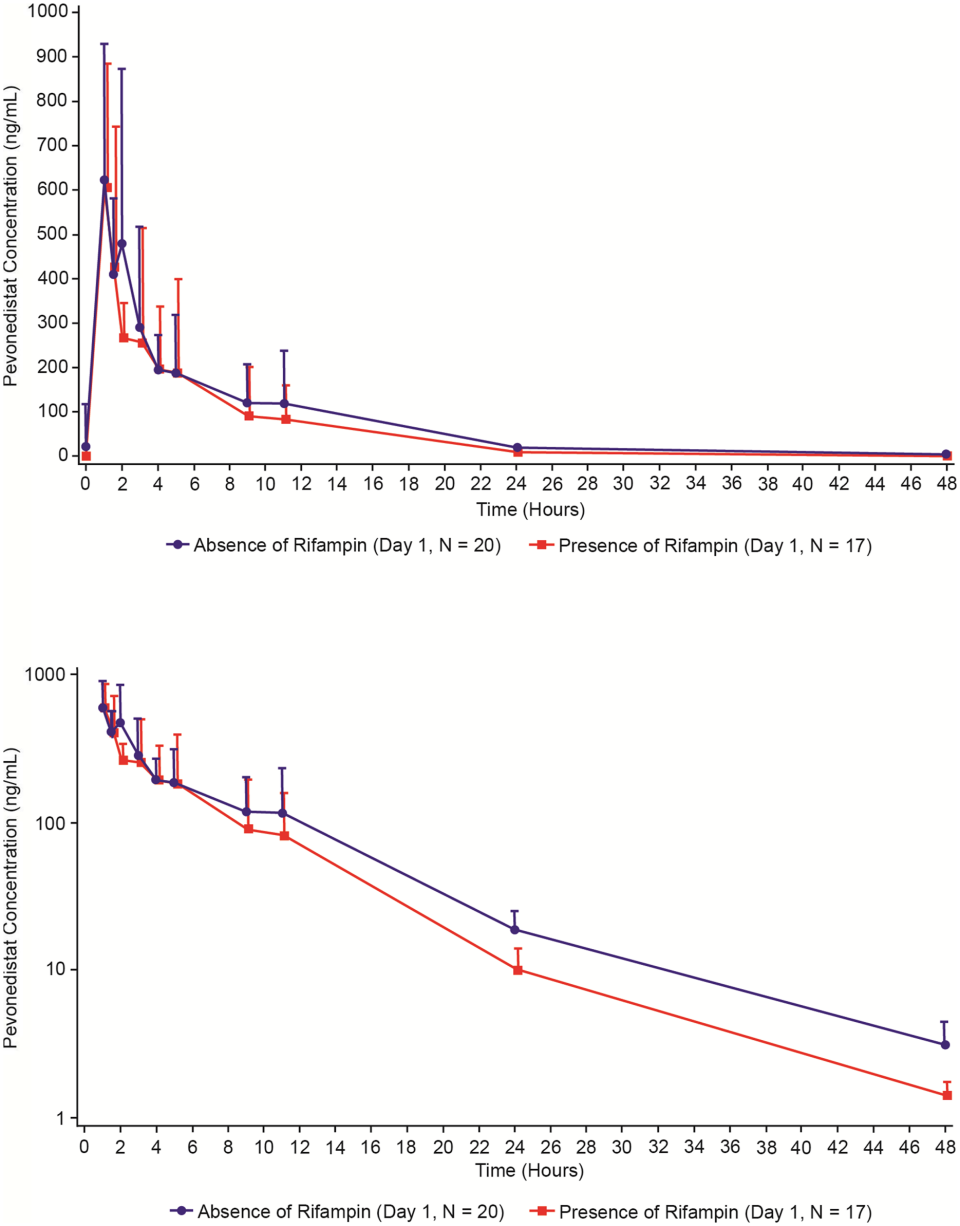
Mean (+ SD) pevonedistat plasma concentration–time profiles in the absence (Day 1) or presence (Day 10) of rifampin (PK population) linear scale (Panel A), semi-log scale (Panel B)

**Fig. 2:**
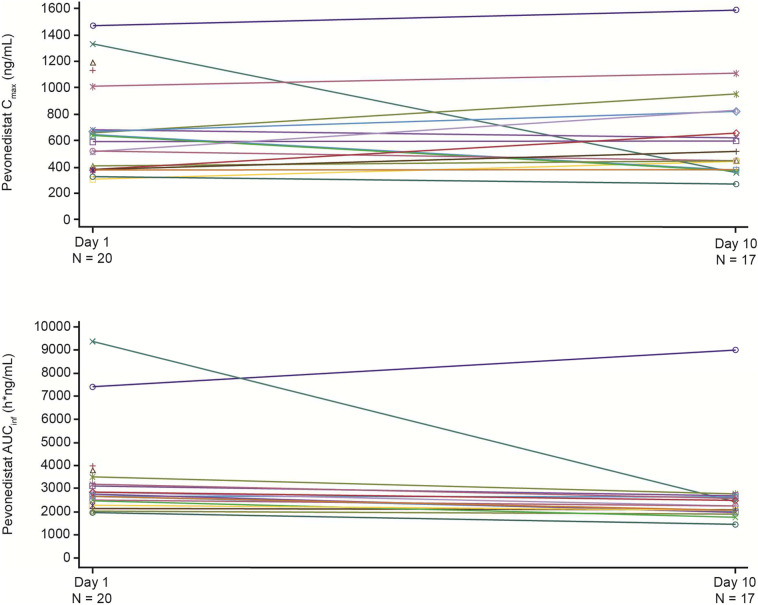
Individual comparisons of AUC_∞_ and C_max_ for pevonedistat in the absence (Day 1) or presence (Day 10) of rifampin (PK population)

Following a single IV infusion of pevonedistat at 50 mg/m^2^, the C_max_ geometric means were 564.1 and 601.7 ng/mL in the presence and absence of rifampin, respectively. Concentration–time profiles of pevonedistat in the presence and in the absence of rifampin are shown in Fig. 1. The mean terminal t_1/2z_ of pevonedistat was 5.7 h and 7.4 h in the presence and in the absence of rifampin, respectively. Summary statistics of pevonedistat PK parameters are provided in Table 2. The geometric mean AUC_∞_ of pevonedistat in the presence of rifampin was 79% of the geometric mean AUC_∞_ of pevonedistat in the absence of rifampin (90% CI: 69.2%–90.2%, *N* = 20 for Day 1 and *N* = 17 for Day 10). Similarly, the geometric mean AUC_last_ of pevonedistat in the presence of rifampin was 78.5% of the geometric mean AUC_last_ of pevonedistat in the absence of rifampin (90% CI: 68.4%–90.1%, *N* = 20 for Day 1 and *N* = 17 for Day 10). The geometric mean C_max_ in the presence and absence of rifampin was similar: 96.2% of the geometric mean C_max_ in the absence (90% CI: 79.2%–117.0%, *N* = 20 for Day 1 and *N* = 17 for Day 10). Table 2 also presents the statistical analysis of the effect of rifampin on pevonedistat PK. Comparison of individual values of pevonedistat C_max_ and AUCs in the presence versus absence of rifampin is illustrated in Fig. 2.

**Table 2:** Summary of single-dose PK parameters of pevonedistat and statistical analysis of effects of rifampin on pevonedistat PK (PK population)

	*N*	C_max_ (ng/mL) geometric mean (CV)	AUC_last_ (h*ng/mL) geometric mean (CV)	AUC∞ (h*ng/mL) geometric mean (CV)	t_1/2z_ (h)mean (SD)	CL (L/h) geometric mean (CV)	V_ss_ (L) geometric mean (CV)
In the absence of rifampin (Day 1)	20	601.7 (52.5%)	2988 (55.9%)	3030 (55.3%)	7.4 (1.4)	33.1 (31.2%)	277.8 (46.2%)
In the presence of rifampin (Day 10)	17	564.1 (53.9%)	2328 (65.6%)	2374 (64.3%)	5.7 (1.4)	41.5 (28.8%)	264.6 (46.0%)

		C_max_ (ng/mL)		AUC_last_ (h*ng/mL)		AUC∞ (h*ng/mL)	
		N	LS mean	N	LS mean	N	LS mean
In the absence of rifampin		20	601.7	20	2988	20	3030
In the presence of rifampin		17	579	17	2345	17	2393
LS mean ratio (90% CI)		0.962 (0.792–1.17)		0.785 (0.684–0.901)		0.790 (0.692–0.902)	

Estimates for each PK parameter were obtained using a mixed-effects model of log (PK parameter) with fixed terms for the rifampin effect and random terms for patient. The CIs are calculated for the difference in the LS means of the ln-transformed AUC_last_, AUC_∞_, or C_max_ values (difference: C1D10-C1D1). Antilogs of the confidence limits for the difference are taken to construct the CIs for the ratio of the geometric means. All parameters presented are in the original scale

*AUC*_*last*_ area under the plasma concentration-time curve from time 0 to time of last quantifiable concentration, *AUC*_*∞*_ area under the plasma concentration-time curve from time 0 extrapolated to infinity, *CL* plasma clearance, *C*_*max*_ observed maximum plasma concentration, *CV* coefficient of variation, *max* maximum, *min* minimum, *PK* pharmacokinetic, *t*_*1/2z*_ terminal half-life, *V*_*ss*_ volume of distribution at steady-state, *LS* least squares

### Safety

 Among 20 patients in the safety population, 16 (80%) patients experienced at least 1 TEAE, and these patients experienced a total of 82 events (Table 3). Nine (45%) patients experienced drug-related TEAEs. Seven (35%) patients experienced grade ≥ 3 TEAEs, and 3 (15%) patients each experienced grade ≥ 3 drug-related TEAEs and serious TEAEs. One patient (5%) died while on study. Two patients experienced a TEAE of hypersensitivity that led to either an interruption of study drug or the drug being permanently withdrawn. The most common TEAEs (≥ 15% of patients) were decreased appetite (4 [20%] patients), fatigue (3 [15%] patients) and diarrhea (3 [15%] patients).

**Table 3:** Overall summary of TEAEs (safety population)

	Patients, n (%) Pevonedistat single dose 50 mg/m^2^ ± rifampin	
	Events	Patients (%)
TEAEs	82	16 (80.0)
Leading to study drug dose delayed	0	0
Leading to study drug dose reduction	0	0
Leading to study drug dose interruption	0	0
Drug-related	14	9 (45.0)
Grade ≥ 3	17	7 (35.0)
Grade ≥ 3 drug related	3	3 (15.0)
Leading to study drug discontinuation	1	1 (5.0)
Serious TEAEs	4	3 (15.0)
Drug related	1	1 (5.0)
Leading to study drug discontinuation	1	1 (5.0)
On-study deaths	1	1 (5.0)

*AE* adverse event, *MedDRA* Medical Dictionary for Regulatory Activities, *TEAE* treatment-emergent adverse event

MedDRA Dictionary (Version 22.0) was used for coding AEs

A TEAE was defined as any AE that occurred after administration of the first dose of study treatment during and up through 30 days after the last dose of study drug

On-study death was defined as a death that occurred between the first dose of study drug and 30 days after the last dose of study drug

## Discussion

Assessment of the effects of intrinsic (e.g., functional capacity of eliminating organs) and extrinsic (e.g., drug–drug interactions) factors on PK plays an essential role in the clinical pharmacology characterization of anticancer agents to inform dosing and administration and risk management in patient populations [19, 20]. Prior to initiating studies to evaluate effects of CYP3A inhibitors or inducers on pevonedistat PK, these concomitant medications were prohibited while patients were receiving pevonedistat in clinical trials. Strong inducers of metabolic enzymes were expected to result in decreased pevonedistat exposures and therefore potential for reduced efficacy.

The selection of the pevonedistat dose of 50 mg/m^2^ to be investigated in this study was based on the recommended dose for clinical investigation determined in a phase 1 dose-finding for pevonedistat as monotherapy: 50 mg/m^2^ dosed on Days 1, 3 and 5 in a 21-day treatment cycle. Pevonedistat concentration–time data from 20 patients were used to assess the effect of rifampin, a strong CYP3A inducer, on pevonedistat single-dose PK. Pevonedistat total plasma clearance of 33 L/h and elimination half-life of approximately 7 h in the absence of rifampin were consistent with previously characterized pevonedistat PK [13, 15]. Rifampin produced an approximately 20% decrease in pevonedistat systemic exposure. These results are not consistent with a major contribution of CYP3A-mediated metabolism to the overall clearance of pevonedistat based on in vitro, clinical mass balance and metabolite profiling [14]. Interestingly, itraconazole, a strong CYP3A/P-glycoprotein inhibitor had no clinically relevant effects on pevonedistat PK [16]. To understand the apparent disconnect between in vitro and clinical observations, a physiologically-based pharmacokinetic (PBPK) model for pevonedistat incorporating metabolic clearance and hepatic uptake was developed to explore the mechanisms underlying its lack of sensitivity to clinically observed drug-drug interactions when administered concurrently with CYP3A inhibitors and inducers [21, 22]. The uptake of pevonedistat was assessed using plated human hepatocytes, and the data were analyzed by incorporating the passive diffusion and active transport components in the PBPK model. The model successfully recovered the lack of change in pevonedistat systemic exposures with concomitant treatment of itraconazole, a strong CYP3A inhibitor. This model was also verified by its ability to recover the observation that pevonedistat plasma exposures are not altered by a clinically meaningful extent in the presence of rifampin. The PBPK modeling suggested a significant induction effect on the hepatic metabolism of pevonedistat in the presence of rifampin. However, systemic exposure of pevonedistat was no longer sensitive to the perturbations of enzyme activity if hepatic uptake was the rate-determining step of pevonedistat clearance.

In conclusion, coadministration of pevonedistat with rifampin, a strong metabolic enzyme inducer resulted in an approximately 20% decrease in systemic exposures of pevonedistat. The 20% decrease in systemic exposure of pevonedistat is not considered to be of clinical relevance in the context of an approximately 30% inter-patient variability in pevonedistat clearance [15]. This result supports the recommendation that no pevonedistat dose adjustment is needed when patients are treated with concomitant strong inducers of PXR-inducible enzymes.

## Supplementary Information

Below is the link to the electronic supplementary material.

Supplementary file1 (DOCX 48 KB)

## Data Availability

The datasets, including the redacted study protocol, redacted statistical analysis plan, and individual participants data supporting the results reported in this article, will be made available within three months from initial request, to researchers who provide a methodologically sound proposal. The data will be provided after its de-identification, in compliance with applicable privacy laws, data protection, and requirements for consent and anonymization.
