# Supplementary material for: Phase 1 study to evaluate the effects of rifampin on pharmacokinetics of pevonedistat, a NEDD8-activating enzyme inhibitor in patients with advanced solid tumors
Source: Invest New Drugs. 2022 Aug 6;40(5):1042–50. doi: 10.1007/s10637-022-01286-8 (PMC9395450; doi:10.1007/s10637-022-01286-8)
Supplement: Supplementary file 1 — Supplementary file1 (DOCX 48 KB) [file 10637_2022_1286_MOESM1_ESM.docx]

**Investigational New Drugs**

**Phase 1 study to evaluate the effects of rifampin on pharmacokinetics of pevonedistat, a NEDD8-activating enzyme inhibitor in patients with advanced solid tumors**

Xiaofei Zhou^1*^, Ulka Vaishampayan^2^, Devalingam Mahalingam^3^, R. Donald Harvey^4^, Ki Young Chung^5^, Farhad Sedarati^1^, Cassie Dong^1^, Douglas V. Faller^1^, Karthik Venkatakrishnan^1,6^, Neeraj Gupta^1^

^1^Takeda Development Center Americas, Inc. (TDCA), Lexington, MA, USA

^2^Karmanos Cancer Institute, Detroit, MI, USA

^3^Northwestern Medical Group, Chicago, IL, USA

^4^Emory University, Atlanta, GA, USA

^5^Prisma Health Cancer Institute/ITOR, Greenville, SC, USA

^6^Current affiliation: EMD Serono Research & Development Institute, Inc., Billerica, MA, USA

***Corresponding author:**

Xiaofei Zhou, PhD

Takeda Development Center Americas, Inc., 40 Landsdowne Street, Cambridge, MA 02139.

[xiaofei.zhou@takeda.com](mailto:neeraj.gupta@takeda.com)

Telephone: +1-617-679-7000

Fax: +1-617-374-7788

**Electronic supplementary material – online only**

**Supplementary Methods**

**Inclusion criteria**

Each patient was required to meet the following inclusion criteria to be enrolled in the study:

1. Were 18 years or older (male or female).
2. Had a histologically or cytologically confirmed metastatic or locally advanced solid tumor that was appropriate for treatment with pevonedistat in combination with either docetaxel or carboplatin + paclitaxel in Part B of this study, or had progressed despite standard therapy, or conventional therapy was not considered effective.
3. Had an Eastern Cooperative Oncology Group (ECOG) performance status of 0 to 1.
4. Had an expected survival of at least 3 months from the date of enrollment in the study.
5. Had recovered (i.e., ≤ grade 1 toxicity) from the effects of prior antineoplastic therapy.
6. Had clinical laboratory values as specified below:

- Hemoglobin ≥ 9 g/dL. Patients may have been transfused to achieve this value.
- Total bilirubin ≤ upper limit of normal (ULN).
- Alanine aminotransferase (ALT), aspartate aminotransferase (AST), and alkaline phosphatase (ALP) ≤ 2.5 times the ULN.
- Calculated creatinine clearance (CrCl) ≥50 mL/min.
- Absolute neutrophil count (ANC) ≥ 1500/mm^3^.
- Platelet count ≥ 100,000/mm^3^.
- Prothrombin time and activated partial thromboplastin time (aPTT) ≤1.5 times the ULN.
- Albumin ≥ 2.7 g/dL.

1. Had suitable venous access for the study-required blood sampling (including PK sampling).
2. For female patients:

- Were postmenopausal for at least 1 year before the screening visit.
- Were surgically sterile.
- If they were of childbearing potential, they and their male partners had agreed to practice 1 highly effective method of contraception and 1 additional effective (barrier) method at the same time, from the time of signing the informed consent through 4 months after the last dose of study drug, or
- Had agreed to practice true abstinence, if in line with the preferred and usual lifestyle of the subject. (Periodic abstinence [e.g., calendar, ovulation, symptothermal, postovulation methods], withdrawal, spermicides only, and lactational amenorrhea were not acceptable methods of contraception. Female and male condoms were not to be used together.)

1. For male patients, even if surgically sterilized (i.e., status postvasectomy):

- Had agreed to practice effective barrier contraception during the entire study treatment period and through 4 months after the last dose of study drug, or
- Had agreed to practice true abstinence, if in line with the preferred and usual lifestyle of the subject. (Periodic abstinence [e.g., calendar, ovulation, symptothermal, postovulation methods], withdrawal, spermicides only, and lactational amenorrhea were not acceptable methods of contraception. Female and male condoms were not to be used together.)

1. Were willing to refrain from donating blood for at least 90 days after their last dose of pevonedistat and (for male patients) were willing to refrain from donating semen for at least 4 months after their last dose of pevonedistat.
2. Voluntary written consent was given before performance of any study‑related procedure not part of standard medical care, with the understanding that consent could be withdrawn by the patient at any time without prejudice to future medical care.

### Exclusion Criteria

Patients who met any of the following exclusion criteria were not to be enrolled in the study:

1. Unable to comply with study visits and procedures including required inpatient confinement.
2. Treated with any systemic antineoplastic therapy or any investigational products within 21 days before the first dose of study treatment.
3. Major surgery within 14 days before the first dose of study treatment or had a scheduled surgery during Part A of the study.
4. Treated with radiation therapy involving ≥ 25% of the hematopoietically active bone marrow.
5. Used strong CYP3A inducers within 2 weeks before the first dose of study drug.
6. Known hypersensitivity or history of severe intolerance or toxicity to chemotherapeutic agents, including known history of severe hypersensitivity reactions to docetaxel (polysorbate 80‑based formulations) for patients to be treated with pevonedistat + docetaxel; history of hypersensitivity to carboplatin for patients to be treated with pevonedistat + carboplatin + paclitaxel; or history of severe hypersensitivity to paclitaxel (Cremophor-based formulations) for patients to be treated with pevonedistat + carboplatin + paclitaxel.
7. Life-threatening illness or serious (acute or chronic) medical or psychiatric illness unrelated to cancer that may have increased the risk associated with trial participation or investigational product administration or may have interfered with the interpretation of trial results or, in the investigator’s opinion, could have potentially interfered with the completion of treatment according to the protocol.
8. An active, uncontrolled infection or severe infectious disease, such as severe pneumonia, meningitis, septicemia, or methicillin-resistant Staphylococcus aureus infection within 2 weeks before dosing.
9. Known human immunodeficiency virus seropositive or known hepatitis B surface antigen seropositive or known or suspected active hepatitis C infection.

*Note:* patients who had isolated positive hepatitis B core antibody (i.e., in the setting of negative hepatitis B surface antigen and negative hepatitis B surface antibody) had to have an undetectable hepatitis B viral load.

1. Persistent diarrhea (grade ≥ 2) lasting > 3 days within 2 weeks before the first dose of study treatment.
2. Known hepatic cirrhosis or severe pre-existing hepatic impairment.
3. Uncontrolled high blood pressure (BP) (i.e., systolic BP > 180 mm Hg, diastolic BP > 95 mm Hg).
4. Left ventricular ejection fraction (LVEF) < 50% within 6 months before study enrollment. If a result within this time frame was unavailable, LVEF must have been determined by echocardiography at screening.
5. Ischemic heart disease with acute coronary syndrome, myocardial infarction, or revascularization (e.g., coronary artery bypass graft, stent) in the past 6 months. However, patients with ischemic heart disease with acute coronary syndrome, myocardial infarction, or revascularization greater than 6 months before screening and who were without cardiac symptoms could be enrolled. Patients who had congestive heart failure (New York Heart Association Class III or IV) or New York Heart Association Class II with recent decompensation requiring hospitalization within 4 weeks before screening and patients with severe pulmonary arterial hypertension were excluded.
6. Arrhythmia (e.g., history of polymorphic ventricular fibrillation or torsade de pointes, permanent atrial fibrillation defined as continuous atrial fibrillation for ≥ 6 months, and persistent atrial fibrillation, defined as sustained atrial fibrillation lasting 7 days and/or requiring cardioversion in the last 4 weeks before screening). However, patients with
   < grade 3 atrial fibrillation for a period of at least 6 months may have enrolled. Grade 3 atrial fibrillation was defined as symptomatic and incompletely controlled medically, or controlled with device (e.g., pacemaker) or ablation, and was excluded. Patients who had paroxysmal atrial fibrillation were permitted to enroll.
7. Prolonged rate corrected QT interval (QTc) ≥ 500 msec, calculated according to institutional guidelines.
8. Implantable cardioverter defibrillator.
9. Cardiac pacemaker set with a fixed heart rate and patients on concomitant medication that may limit increase in heart rate in response to hypotension (e.g., high-dose beta blocker).
10. Moderate to severe aortic stenosis, moderate to severe mitral stenosis, or other valvulopathy (ongoing).
11. Known moderate to severe chronic obstructive pulmonary disease, interstitial lung disease, pulmonary fibrosis, or pulmonary arterial hypotension.
12. Female patients who were lactating and breastfeeding or who had a positive serum pregnancy test during the screening period or a positive urine pregnancy test on Day 1 before the first dose of study drug.
13. Female patients who intended to donate eggs (ova) during the course of this study or 4 months after receiving their last dose of study drug.
14. Male patients who intended to donate sperm during the course of this study or 4 months after receiving their last dose of study drug.
15. Required chronic treatment with BCRP inhibitors.
